# Curiosities in Medical Teaching

**Published:** 1881-02

**Authors:** 


CURIOSITIES IN MEDICAL TEACHING.—We are informed that much of the instruction given by a professor of Materia Medica in one of the homœopathc medical colleges in this city, is imparted in the form of questions to be answered by the class. The following are a few specimens of the questions and answers, taken verbatim by a student during attendance on one of the regular lecture hours in that school:

1. *Professor.*—What is the proper remedy for *mania*, with desire to cut and tear things? *Student.*—Veratrum.

2. *P.*—For pain in the abdomen flying upward? *S.*—Bryony.

3. *P.*—For *tip* of the *nose*, red and hot, particularly in warm weather? *S.*—Belladonna.

4. *P.*—For emaciation of upper part of the body while the lower part is enormously swollen? *S.*—Lycopodium.

5. *P.*—For child that dislikes to be washed or bothered? *S.*—Sulphur.

6. *P.*—For easily biting cheek, in chewing? *S.*—Ignatia.

7. *P.*—For mumps on the *left* side? *S.*—Rhus Tox.

8. *P.*—For mumps on the right side? *S.*—Mercurius.

9. *P.*—For constantly dreaming of dead persons? *S.*—Arsenicum.

10. *P.*—For constantly dreaming of robbers? *S.*—Ignatia.

These are fair samples of the whole.

